# Markers of Cardiac Autonomic Function During Consecutive Day Peak Exercise Tests in People With Myalgic Encephalomyelitis/Chronic Fatigue Syndrome

**DOI:** 10.3389/fphys.2021.771899

**Published:** 2021-12-14

**Authors:** Maximillian J. Nelson, Jonathan D. Buckley, Rebecca L. Thomson, Clint R. Bellenger, Kade Davison

**Affiliations:** ^1^Alliance for Research in Exercise, Nutrition and Activity (ARENA), UniSA Allied Health and Human Performance, University of South Australia, Adelaide, SA, Australia; ^2^Adelaide Medical School and Robinson Research Institute, University of Adelaide, Adelaide, SA, Australia

**Keywords:** Myalgic Encephalomyelitis, Chronic Fatigue Syndrome, autonomic function, heart rate, fatigue, heart rate variability

## Abstract

Patients with Myalgic Encephalomyelitis/Chronic Fatigue Syndrome (ME/CFS) have been shown to exhibit altered ventilatory characteristics on the second of two progressive maximal cardiopulmonary exercise tests (CPET) performed on consecutive days. However, maximal exercise can exacerbate symptoms for ME/CFS patients and cause significant post-exertional malaise. Assessment of heart rate (HR) parameters known to track post-exertional fatigue may represent more effective physiological markers of the condition and could potentially negate the need for maximal exercise testing. Sixteen ME/CFS patients and 10 healthy controls underwent a sub-maximal warm-up followed by CPET on two consecutive days. Ventilation, ratings of perceived exertion, work rate (WR) and HR parameters were assessed throughout on both days. During sub-maximal warm-up, a time effect was identified for the ratio of low frequency to high frequency power of HR variability (*p*=0.02) during sub-maximal warm-up, and for HR at ventilatory threshold (*p*=0.03), with both being higher on Day Two of testing. A significant group (*p*<0.01) effect was identified for a lower post-exercise HR recovery (HRR) in ME/CFS patients. Receiver operator characteristic curve analysis of HRR revealed an area under the curve of 74.8% (*p*=0.02) on Day One of testing, with a HRR of 34.5bpm maximising sensitivity (63%) and specificity (40%) suggesting while HRR values are altered in ME/CFS patients, low sensitivity and specificity limit its potential usefulness as a biomarker of the condition.

## Introduction

Myalgic Encephalomyelitis/Chronic Fatigue Syndrome (ME/CFS) is a chronic condition of unexplained onset, characterised by both physical and mental fatigue, muscle and joint pain and increased levels of post-exertional malaise when compared with healthy individuals (Fukuda et al., 1994). The prevalence of ME/CFS has been estimated at 0.8 to 3.3% of the population (Johnston et al., 2013). The condition represents a significant challenge to patients and healthcare providers given that many patients are unable to maintain their occupation and there is no widely accepted treatment (Carruthers et al., 2003).

In addition to there being no accepted treatment for ME/CFS, difficulties also exist with its diagnosis. Many studies have attempted to identify a single, objective biomarker to aid with diagnosis (Demitrack and Crofford, 1998; Barnden et al., 2011; Frith et al., 2012); however, to date, none has been identified. As a result, diagnosis has been performed based on clinical criteria which aim to confirm a set of core symptoms and exclude other factors which might otherwise explain these symptoms. These clinical criteria have typically required the presence of fatigue exacerbated by exercise, sore throat, headaches and unrefreshing sleep, among other symptoms (Fukuda et al., 1994; Carruthers et al., 2003).

Recent studies have investigated the effects of consecutive day maximal cardiopulmonary exercise tests (CPET) to identify post-exertional, fatigue-induced biomarkers that can discriminate between ME/CFS patients and controls (Vanness et al., 2007; Vermeulen et al., 2010; Keller et al., 2014). Multiple studies (Vanness et al., 2007; Snell et al., 2013; Keller et al., 2014) have identified that ME/CFS patients experience an earlier onset of ventilatory threshold (VT) on the second day of consecutive day maximal exercise testing, a change that is not present in healthy controls. This finding was confirmed in a recent meta-analysis (Lim et al., 2020). Although this earlier onset of VT may represent an objective biomarker of ME/CFS, requiring patients with ME/CFS to complete graded exercise tests to exhaustion on two consecutive days may exacerbate fatigue and is therefore not ideal for assisting with the diagnosis of ME/CFS. As a result, there is interest in establishing a tool to aid in the diagnosis of ME/CFS which does not exacerbate symptoms.

A number of studies have investigated the presence of autonomic dysfunction in ME/CFS patients (De Becker et al., 1998; Newton et al., 2007). Since heart rate (HR) parameters have been shown to reflect autonomic function, research has focused on the use of HR to detect differences in autonomic balance in ME/CFS patients. Previously studied HR parameters which have been used to evaluate autonomic regulation in patients with ME/CFS include resting HR (RHR; De Becker et al., 1998; Newton et al., 2009), HR variability (HRV; Yataco et al., 1997; Frith et al., 2012) and HR recovery (HRR; Fulcher and White, 2000). A recent meta-analysis of all published research on the topic (Nelson et al., 2019a) found that ME/ CFS patients exhibited multiple HR alterations, including increases in resting RHR, HRR, HR response to head up tilt testing, average HR during a 24h period, the ratio of low frequency power to high frequency power of resting HRV (LF/HF), the high frequency (HF) portion of HRV and decreases in maximal HR and HR at anaerobic threshold. Taken together, these results provide clear evidence of increased sympathetic and decreased parasympathetic cardiac modulation in patients with ME/CFS compared with healthy controls but did not identify any parameters which could aid in the diagnosis of the condition (Nelson et al., 2019a).

Although HR parameters as investigated to date have not been useful biomarkers of ME/CFS, multiple parameters [e.g. HRV, HRR and maximal rate of heart rate increase at exercise onset (rHRI)] have been shown to be altered as a result of training-induced fatigue in athletic populations (Bellenger et al., 2016). In particular, rHRI has shown promise as a marker of post-exertional fatigue in male (Nelson et al., 2020) and female (Nelson et al., 2017) athletes but has never been assessed in ME/CFS patients. It is therefore possible that rHRI and other HR parameters assessed as part of CPET may be able to identify changes in autonomic cardiac function which result from post-exertional malaise within ME/CFS, and thereby act as a biomarker of the condition without the need for maximal exercise on the second day of testing. Accordingly, this study aimed to determine whether cardiac autonomic modulation exhibits a differing response to consecutive day CPET testing in ME/CFS patients with a view to determining if any HR parameters could represent an objective biomarker of the condition.

## Materials And Methods

**Figure 1:**
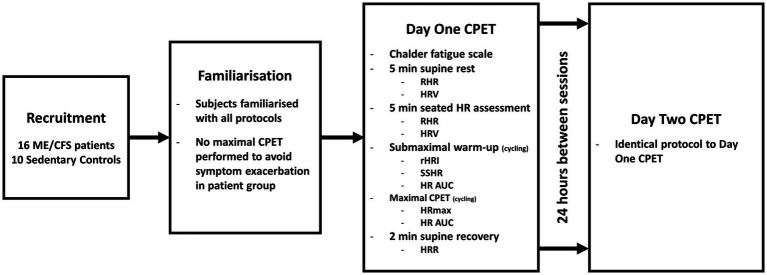
Diagram of study design. *ME/CFS*, Myalgic Encephalomyelitis/Chronic Fatigue Syndrome; *CPET*, cardiopulmonary exercise test; *min*, minutes; *RHR*, resting heart rate; *HRV*, heart rate variability; *HR*, heart rate; *rHRI*, maximal rate of heart rate increase; *SSHR*, steady state heart rate; *AUC*, area under the curve; *HRmax*, maximal heart rate; and *HRR*, heart rate recovery.

This study represents a sub-analysis of a larger study (Nelson et al., 2019b). Briefly, following familiarisation, participants performed a sub-maximal warm-up followed by a CPET to volitional exhaustion on two consecutive days. Parameters of ventilation, performance, rating of perceived exertion and HR were assessed throughout the protocol. Aspects of the protocol relating to the collection of ventilatory and performance data have been reported elsewhere (Nelson et al., 2019b). A brief overview of participants and experimental procedures with additional detail relating to the assessment of HR parameters is found in the following sections. The study design is illustrated in Figure 1.

### Participants

Sixteen ME/CFS patients [nine female; age (mean±SD) 50.3±12.5years, body mass index 25.9±5.3kg/m2] were recruited *via* specialist clinics and ME/CFS support groups from the Adelaide, South Australia Greater Metropolitan area. Ten healthy participants (five female, age 49.8±13.7years, BMI 24.6±3.0kg/m2) matched to participants were recruited to act as controls using convenience sampling. Participants were required to be between the ages of 18–65years, and ME/CFS patients had to have been previously diagnosed with ME/CFS based on one of three widely accepted diagnostic criteria: (1) 1994 Centres For Disease Control and Prevention [CDC 1994 – also known as the ‘Fukuda’ criteria (Fukuda et al., 1994)], (2) 2003 ‘Canadian’ Consensus Criteria (Carruthers et al., 2003) or (3) 2011 International Consensus Criteria (Carruthers et al., 2011). All participants were required to be sedentary (<150min of moderate physical activity per week) and were excluded if they were taking any medication or had any known medical conditions (excluding ME/CFS) which could alter HR (e.g. beta-blockers and anti-depressants/postural orthostatic tachycardia syndrome). Experimental procedures were approved by the University of South Australia Human Research Ethics Committee and conformed to the Declaration of Helsinki, with all participants providing written informed consent prior to participating.

### Experimental Procedures

Participants first attended the laboratory for an initial habituation session, during which they were familiarised with the laboratory and with the questionnaires and procedures to be used during the study. Following the familiarisation session, participants returned to the laboratory at least 24h later for the first of two exercise testing sessions. Each session had an identical protocol and was performed on consecutive days. Participants first completed the Chalder Fatigue Scale (23) and then were fitted with a HR monitor (RS800CX, Polar Electro Oy, Kempele, Finland) and given 10min of supine rest, during which data to determine RHR and resting HRV were collected. Participants were then seated on a bicycle ergometer (Ergoselect 200, Ergoline GmbH, Bitz, Germany) and fitted with the breathing valve (Hans-Rudolph Inc., Shawnee, Kansas) connected to an indirect calorimetry system (TrueOne 2,400, Parvo Medics, East Sandy, Utah). Following 4–6 minutes of seated rest on the ergometer which allowed for the calculation of seated HR and HRV parameters, participants were then instructed to commence a sub-maximal warm-up, which consisted of cycling at a self-selected cadence for 5 minutes at 40 W for males and 30 W for females. Following the initial 5minutes of steady state exercise, the work rate was increased by 5W increments every 20s, until volitional exhaustion. All participants were given frequent verbal encouragement throughout the incremental portion of the test to help elicit a maximal effort (Andreacci et al., 2002). Immediately following the cessation of exercise, participants were assisted to dismount the cycle ergometer and lay supine for 2minutes for assessment of HRR. Following the initial exercise test, participants returned to the laboratory at the same time on the following day, and the protocol was repeated in an identical fashion.

### Heart Rate Parameters

All HR data were downloaded as R-R intervals to Polar Protrainer 5 software (Polar Electro Oy, Kempele, Finland), where artefacts or ectopic heartbeats were removed using the software’s automatic data filtering function. RHR was defined as the average HR during the last 2minutes of pre-exercise supine rest. rHRI was quantified by fitting a 5-parameter sigmoidal curve to R-R interval data recorded during the rHRI assessment, as described elsewhere (Bellenger et al., 2017) using data from the 30s prior to exercise onset, and the subsequent first 3minutes of steady state cycling during the sub-maximal warm-up (Nelson et al., 2020). rHRI has previously been shown to be reliable, with a coefficient of variation of 6.3% (Nelson et al., 2014). Steady state HR (SSHR) was extracted as the average HR during the final 30s of the sub-maximal warm-up. HRR was calculated as both the decrease in HR (beats/min) in the first minute of post-exercise supine rest (HRR Δ1) and the absolute HR after 1minute of post-exercise supine rest (HRR 1'; Boullosa et al., 2014; Del Rosso et al., 2017). To provide a marker of the psycho-physiological stress influence on HR during exercise (Medeiros et al., 2016), the area under the curve (AUC) for the relevant HR curve was calculated using a custom-made spreadsheet (Microsoft Excel 16, Microsoft Corporation, Redmond, Washington) during the 5minutes of steady state cycling and during the incremental portion of the CPET. HRV parameters were calculated by exporting R-R data during the last 2minutes of pre-exercise supine rest in addition to the last 2minutes of pre-exercise seated rest (supine and seated postures analysed separately), and for non-frequency domain analyses, during the 5minutes of steady state exercise to HRV analysis software (Kubois HRV analysis, version 2.0 beta 1, Biomedical Signals Analysis Group, University of Kuopio, Finland), where secondary screening of artefacts and ectopic heartbeats took place. Frequency domain analyses were defined as the spectral power calculated using a Fast Fourier Transform (Welch’s periodgram: 256s window with 50% overlap), with the low frequency power (LFP) band defined as 0.04–0.15Hz, and the high frequency power (HFP) band defined as 0.15–0.4Hz, in addition to, the ratio of LFP to HFP (LF/HF) (Malik et al., 1996). R-R intervals (RR) were analysed to provide a sympatho-vagal index (Medeiros et al., 2018). In addition, the time-domain parameter of the root-mean-square difference of successive normal R-R intervals (RMSSD) was calculated to provide an additional measure of parasympathetic HR modulation (Buchheit, 2014). RMSSD has been shown to have moderate to good day-to-day reliability for supine and poor to good day-to-day reliability for standing postures (Medeiros et al., 2021).

### Statistical Analysis

Statistical analysis was performed using IBM SPSS Statistics 21 (IBM Corporation, Armonk, NY). All data were checked for normality of distribution using the Shapiro-Wilk test prior to analysis. Unpaired t-tests were used to determine if there were any differences at baseline (Day One) between patients and controls for any dependant variables. To determine the effect of post-exertional malaise on the dependant variables, two-way repeated measures ANOVA was performed to identify any main effects of group (patient or control) and time (Day One or Day Two). Interaction effects of group×time were generated as part of the two-way ANOVA. Where significant main effects were identified, estimated marginal means were assessed to determine where those differences occurred. To determine if any of the assessed parameters represent a useful tool to aid in differentiating between ME/CFS patients and controls, receiver operator characteristic (ROC) analysis was conducted on any physiological variables which demonstrated a significant group or group×time interaction in order to compute sensitivity and specificity of these variables for differentiating ME/CFS participants from controls. Statistical significance was set at *p*<0.05. All data are presented as mean±standard deviation (*SD*).

## Results

Results for HR parameters are provided in Table 1. All participants produced a valid maximal effort during exercise testing (Nelson et al., 2019b). ME/CFS patients reported higher scores than controls on the Chalder Fatigue Scale on Day One (ME/CFS 20.3±6.8, Controls 11.2±0.6, *p*<0.01), with a significant group×time interaction effect following Day Two (ME/CFS 25.3±7.0, Controls 11.2±0.6) due to a greater increase in their scores compared with controls (*p*<0.01).

**Table 1:** Heart rate parameters obtained during consecutive days of maximal exercise testing.

Variable	Controls Day 1	ME/CFS Day 1	Controls Day 2	ME/CFS Day 2	‘Group’ effect value of *p*	‘Time’ effect value of *p*	‘Group’×‘Time’ interaction value of *p*
Supine							
HR (bpm)	69.5 (8.4)	74.0 (12.1)	70.1 (10.9)	72.0 (12.0)	0.471	0.607	0.350
RR (ms)	871.0 (98.7)	846.1 (145.4)	870.75 (128.5)	847.8 (149.6)	0.659	0.928	0.938
LFP (ms^2^)	616.3 (607.9)	631.2 (737.6)	710.9 (1171.9)	602.3 (558.1)	0.861	0.842	0.709
HFP (ms^2^)	207.1 (237.7)	221.1 (194.3)	272.3 (340.7)	245.3 (305.4)	0.949	0.271	0.609
LF/HF	4.66 (5.68)	3.95 (3.56)	4.35 (2.75)	4.82 (5.29)	0.935	0.783	0.551
RMSSD (ms)	21.0 (12.6)	22.6 (10.3)	23.5 (14.6)	20.5 (10.2)	0.877	0.872	0.141
Seated							
HR (bpm)	80.0 (8.4)	83.1 (12.3)	80.2 (10.4)	81.9 (11.3)	0.556	0.812	0.740
RR (ms)	771.0 (81.4)	753.3 (136.8)	764.8 (99.2)	767.4 (116.3)	0.874	0.784	0.430
LFP (ms^2^)	365.5 (331.1)	529.3 (429.0)	332.7 (212.7)	604.2 (557.6)	0.199	0.688	0.308
HFP (ms^2^)	221.2 (321.2)	428.1 (774.5)	195.0 (265.6)	223.6 (349.4)	0.499	0.313	0.433
LF/HF	4.12 (3.50)	5.64 (5.10)	3.434 (3.771)	6.73 (7.91)	0.222	0.874	0.491
RMSSD (ms)	17.6 (9.1)	20.7 (14.3)	17.4 (9.0)	18.8 (9.4)	0.591	0.509	0.600
Steady state							
HR (bpm)	100.6 (11.1)	106.5 (12.6)	101.0 (12.7)	104.5 (14.2)	0.331	0.716	0.556
RR (ms)	607.2 (70.9)	594.8 (83.2)	611.1 (77.8)	590.9 (82.1)	0.304	0.898	0.637
rHRI (bpm/s)	3.481 (3.21)	2.315 (2.68)	2.75 (2.10)	2.09 (1.96)	0.329	0.243	0.538
RMSSD (ms)	9.9 (5.7)	8.4 (4.8)	9.6 (6.1)	7.8 (4.8)	0.422	0.514	0.812
AUC	29447.9 (3095.1)	31047.1 (4164.1)	29386.5 (3665.3)	29636.3 (2638.0)	0.520	0.251	0.291
Ventilatory threshold							
HR (bpm)	122.4 (13.2)	124.0 (18.3)	120.8 (14.2)	117.4 (17.6)	0.892	0.030	0.173
Maximal exercise							
HR (bpm)	170.3 (10.0)	167.8 (20.0)	170.9 (11.0)	167.0 (15.7)	0.600	0.936	0.666
AUC	245324.3 (73468.9)	236193.1 (155736.3)	245790.6 (72822.6)	220060.2 (127967.4)	0.730	0.449	0.423
HRR 1' (bpm)	127.1 (15.4)	134.6 (22.5)	126.8 (15.1)	130.0 (19.1)	0.466	0.372	0.422
HRR Δ1^a^ (bpm)	43.2 (7.9)	33.2 (6.9)^a^	44.1 (9.3)	36.9 (8.8)	0.007	0.151	0.373

*Values are mean (SD), asignificant difference between ME/CFS and controls at Day 1 (*p*<0.05), ME/CFS, Myalgic Encephalomyelitis/Chronic Fatigue Syndrome; HR, heart rate; bpm, beats per minute; RR, interval between R waves; LFP, low frequency power from spectral analysis; HFP, high frequency power from spectral analysis; LF/HF, ratio between low frequency and high frequency power from spectral analysis; RMSSD, root-mean-square difference of successive normal R-R intervals from time-domain analysis; rHRI, maximal rate of increase in heart rate; sec, second; AUC, area under the curve for time (sec) vs HR (bpm); and HRR, post-exercise heart rate recovery*.

Significant effects of time for HR at VT were identified, with estimated marginal means indicating that values were lower on Day Two (*p*=0.03, Table 1). In addition, a significant effect of group was found for HRR Δ1 (*p*<0.01), with HRR values being lower for ME/CFS patients, particularly on Day One (Table 1). There were no group, time or group×time interaction effects for any other HR parameters in supine or seated postures, or during steady state exercise (*p*>0.05, Table 1).

ROC analysis of HRR values from Day One and Day two of testing showed areas under the curve of 74.8% (*p*=0.02, standard error: 9.4%) and 70.3% (*p*=0.09, standard error 11.0%) and found optimal sensitivity for differentiating between patients and controls with HRR values less than 34.5bpm using HRR values from Day One (sensitivity=63%, specificity=40%) and 37.5bpm using HRR values from Day Two (sensitivity=69%, specificity=30%) indicating a diagnosis of ME/CFS.

## Discussion

The main finding of the present study was that while ME/CFS patients demonstrated increased fatigue both physiologically (i.e. HR at VT) and perceptually on the second bout of maximal exercise 24h following the initial bout, there were no differential changes in any HR other parameters. This suggests that HR-based measures of autonomic function appear to be unaffected by post-exertional malaise and cannot therefore contribute to this aspect of ME/CFS diagnosis. rHRI, which has been shown to track closely with training-induced fatigue in athletes, and never been previously assessed in ME/CFS patients, was not different at baseline nor following CPET. HRR Δ1 was found to be lower for ME/CFS patients; however, the results of ROC analysis for diagnostic sensitivity suggest it is unlikely to be a useful method for differentiating between ME/CFS patients and controls.

rHRI was not different between patients and controls and did not change with consecutive days of maximal exercise testing in either group. In previous studies of rHRI in well-trained individuals (Nelson et al., 2014, 2017; Bellenger et al., 2015, 2017), rHRI was slowed in response to exercise-induced fatigue and increased in response to physiological adaptation. In the current study, following the induction of post-exertional malaise in ME/CFS patients *via* the first maximal test, there was no change in rHRI. However, rHRI has previously tracked exercise-induced changes in performance, but in the current study, there were no changes in performance from Day One to Day Two (evidenced by no change in peak HR, peak WR and peak RER; Nelson et al., 2019b), despite patients reporting a significant exacerbation of fatigue symptoms (evidenced by an increase in the Calder Fatigue Scale score). This suggests that patients with ME/CFS suffer from a form of pathological fatigue/malaise that manifests independently of actual muscular fatigue [i.e. an inability to maintain a given power output (Edwards, 1983) or a change in physical task or mechanical performance (Kluger et al., 2013)]. It appears that rHRI may track changes in performance at the muscle resulting from post-exertional fatigue, but not the pathological fatigue that was present in ME/CFS patients in the current study. While the main study these data were derived from Nelson et al. (2019b) reported altered ventilatory kinetics, there was no detectable performance change, suggesting no fatiguing effect on muscle or overall cardiorespiratory function. The lack of change in rHRI on Day Two coupled with no change in maximal exercise performance is consistent with a recent study on changes in rHRI resulting from anaerobic fatiguing interventions (D’unienville et al., 2021), where authors suggested that the fatigue-induced slowing in rHRI appears to be primarily mediated by peripheral inputs which arise from fatigued skeletal muscle, rather than factors within the central nervous system. While there is evidence of metabolic abnormalities in skeletal muscle of ME/CFS patients (Mccully and Natelson, 1999; Jones et al., 2010, 2012), recent studies have suggested that the condition may result from pathological alterations in the function of the central nervous system (Siemionow et al., 2004; Chen et al., 2008; Meeus et al., 2013). This might explain why differences in exercise performance and rHRI were not detected in this study.

Apart from rHRI, this study assessed multiple HR parameters during both rest and exercise but found very few differences between ME/CFS patients at baseline (Day One) or in response to the consecutive day maximal exercise testing protocol. HRR Δ1 was found to be affected by a significant effect of group indicating HRR values were significantly lower for patients than controls overall but was unchanged following the 2day testing protocol. However, the large overlap in HRR values between patients and controls suggests it is unlikely to be a useful parameter to aid in the diagnosis of ME/CFS. ROC analysis revealed only moderate levels of sensitivity and specificity for HRR Δ1 assessed on either day of testing (Day One, sensitivity=63%, specificity=40%; Day Two: sensitivity=69%, specificity=30%). Although this suggests that HRR Δ1 may have low level capabilities to differentiate between ME/CFS patients and controls, the results from the ROC analysis are weaker than those seen in previous studies which have aimed to determine if HR parameters can be used to differentiate between patients and controls, including Frith et al. (2012) who found that a combination of resting blood pressure variability and HRV values may be useful for differentiating between ME/CFS patients and healthy controls (sensitivity 77%, specificity 53%). Importantly, it should be acknowledged that other HRR parameters which were not assessed within the current study may be able to differentiate between patients and controls more sensitively. This study only included analysis of HRR assessed during the first minute following exercise and was recorded in a supine position immediately after dismounting the bicycle ergometer. The change in posture immediately prior to HRR analysis may have affected the HRR values during the recording period, so future research should consider exploring additional recovery postures and parameters (e.g. longer durations of recovery).

Apart from the differences in HRR and HR at VT, there were no differences for any HR parameters between patients and controls nor were there any changes in HR parameters from Day One to Day Two of testing. Recent meta-analysis (Nelson et al., 2019a) has found evidence of a resting sympathetic hyperactivity in ME/CFS patients, characterised by an increase HFP and a decreased LFP; however, this finding was not repeated in the current study. Potentially, the altered resting autonomic balance in ME/CFS patients seen in some previous studies was due to ME/CFS patients being deconditioned in comparison with their healthy counterparts, as research has shown that increased physical fitness causes an increased resting parasympathetic tone (Carter et al., 2003). It is important to acknowledge, however, that ME/CFS patients and controls were well matched within the current study. As was previously shown (Nelson et al., 2019b), there were no differences between patients and controls for peak V̇O_2_, peak WR (both reported previously) RHR, SSHR or peak HR, suggesting that the patients and controls were well matched for their general fitness levels. Previous literature suggests that ME/CFS patients may be affected by deconditioning (De Lorenzo et al., 1998) or kinesiophobia (Nijs et al., 2004, 2012). However, as the patients and controls within the current study were similar in peak V̇O_2_ and peak WR (Nelson et al., 2019b), this suggests deconditioning is unlikely to have impacted HR parameters assessed within this study.

This study is limited by a possible selection bias as a result of the consecutive day maximal exercise tests used within the study. Given the potential for symptom exacerbation as a result of the consecutive day maximal exercise tests, patients with severe ME/CFS may be less likely to volunteer, whereas patients with mild-moderate ME/CFS may be more likely. Anecdotally, none of the included participants classified themselves as a severe sufferer of the condition, so the inclusion of patients with a more severe form of ME/CFS may have produced a different result. Future research should attempt to include sufferers with severe ME/CFS in the study design; however, the authors acknowledge the potential ethical difficulties associated with this idea, given the potential for drastic symptom exacerbation for these patients. Further, the lack of reliability in some HR parameters cannot be dismissed. While some parameters (e.g. rHRI) have been shown to have good day-to-day reliability (Nelson et al., 2014), others (e.g. HRV frequency analysis) have been shown to have questionable reliability despite it being a well-accepted tool for investigating such cardiac autonomic parameters (Cipryan and Litschmannova, 2013) and this is particularly relevant given the small sample size within this study. Future studies should consider the implications of the varying reliability of HR parameters on their sample size in the context of the patient group within which they are working.

## Conclusion

Heart rate markers of autonomic function were unchanged in ME/CFS patients in the presence of post-exertional malaise, induced by maximal CPET on consecutive days. HR parameters assessed during this protocol are unlikely to represent a useful biomarker of the condition.

## Data Availability Statement

The datasets presented in this article are not readily available because some parts of the data are related to other articles under review, for which data will be available once accepted for publication. Requests to access the datasets should be directed to max.nelson@unisa.edu.au.

## Ethics Statement

The studies involving human participants were reviewed and approved by the University of South Australia. The patients/participants provided their written informed consent to participate in this study.

## Author Contributions

MN designed the study, collected the data, analysed and interpreted the data, and was the primary author of the manuscript. JB, RT, CB, and KD designed the study, interpreted the data, and drafted the final manuscript. All authors have read and approved the final manuscript.

## Funding

Researcher MN was supported by an Australian Government Research Training Program Scholarship; however, this funding body had no role in the design of the study, collection, analysis or interpretation of data.

## Conflict of Interest

JB was the inventor of the rHRI technology described in this article and is an employee of the University of South Australia and has assigned his rights in the technology to the University. The rHRI technology has been patented by the University of South Australia, which is seeking to commercialise it. Researchers MN, CB and KD are also employees of the University of South Australia, while RT is an adjunct researcher at the University.

## Publisher’s Note

All claims expressed in this article are solely those of the authors and do not necessarily represent those of their affiliated organizations, or those of the publisher, the editors and the reviewers. Any product that may be evaluated in this article, or claim that may be made by its manufacturer, is not guaranteed or endorsed by the publisher.
